# Radiographic findings useful for diagnosis of primary chest wall lymphoma without preceding pleural disease: A case report

**DOI:** 10.1002/rcr2.1019

**Published:** 2022-08-15

**Authors:** Masanori Tanaka, Daichi Fujimoto, Hiroaki Akamatsu, Hiromitsu Sumikawa, Nobuyuki Yamamoto

**Affiliations:** ^1^ Internal Medicine III Wakayama Medical University Wakayama Japan; ^2^ Department of Radiology National Hospital Organization Kinki‐Chuo Chest Medical Center Osaka Japan

**Keywords:** bone, computed tomography, fluorodeoxyglucose‐positron emission tomography, lymphoma, primary chest wall tumour

## Abstract

A 70‐year‐old man with no history of pleural diseases had a dumbbell‐shaped chest wall mass extending from the thoracic cavity to the spinal canal at the intervertebral foramen without bone destruction. Computed tomography revealed a positive a ‘pleural sandwich sign’, where the intercostal artery was enveloped by the mass. A high maximum standard uptake value was noted on fluorodeoxyglucose‐positron emission tomography. No lesions were found in areas other than the chest wall. CT‐guided biopsy was performed and he was diagnosed with primary chest wall lymphoma. This case report suggests that these radiographic findings may be helpful for diagnosing chest wall lymphomas even in patients without prior pleural disease.

## Graphical abstract

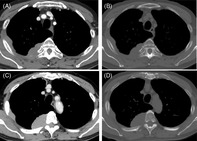

## INTRODUCTION

Primary chest wall tumours account for approximately 5% of all chest tumours.^1^ Out of which, 50%–80% of primary chest wall tumours are malignant.^2^, ^3^, ^4^ They originate from diseases of the bone, cartilage, blood or soft tissue (muscles, vessels and nerves).^3^

Primary chest wall lymphoma without preceding pleural disease is rare and difficult to diagnose. Incisional or computed tomography (CT)‐guided biopsies are the recommended diagnostic modalities due to the high false‐negative rate of fine needle aspiration biopsy.^5^ Additionally, flow cytometry (FCM) may also be used as an auxiliary diagnostic method because some malignant lymphomas lack clear morphological changes seen in biopsies. Since FCM requires live cells without formalin fixation, it is difficult to examine without suspicion of malignant lymphoma before a biopsy. Therefore, primary chest wall lymphoma without prior pleural disease should be considered as a differential diagnosis based on radiographic findings prior to biopsy to make a proper diagnosis. However, there are currently no reports summarizing the possible imaging features of primary malignant lymphoma of the chest wall without prior pleural disease.

We herein report a patient with notable radiographic findings indicative of a primary chest wall lymphoma. A literature review of imaging was also provided.

## CASE REPORT

A 70‐year‐old man presented to our department with a one‐month history of persistent right back pain. He had no history of pleural tuberculosis, chronic pyothorax or dust exposure. On physical examination, no tenderness or swelling of the chest wall and palpable systemic lymphadenopathy were noted. A band‐shaped sensory disturbance was observed from the right anterior chest to the lateral chest. Laboratory examination showed that his soluble interleukin 2 receptor level was 981 U/ml.

**FIGURE 1:**
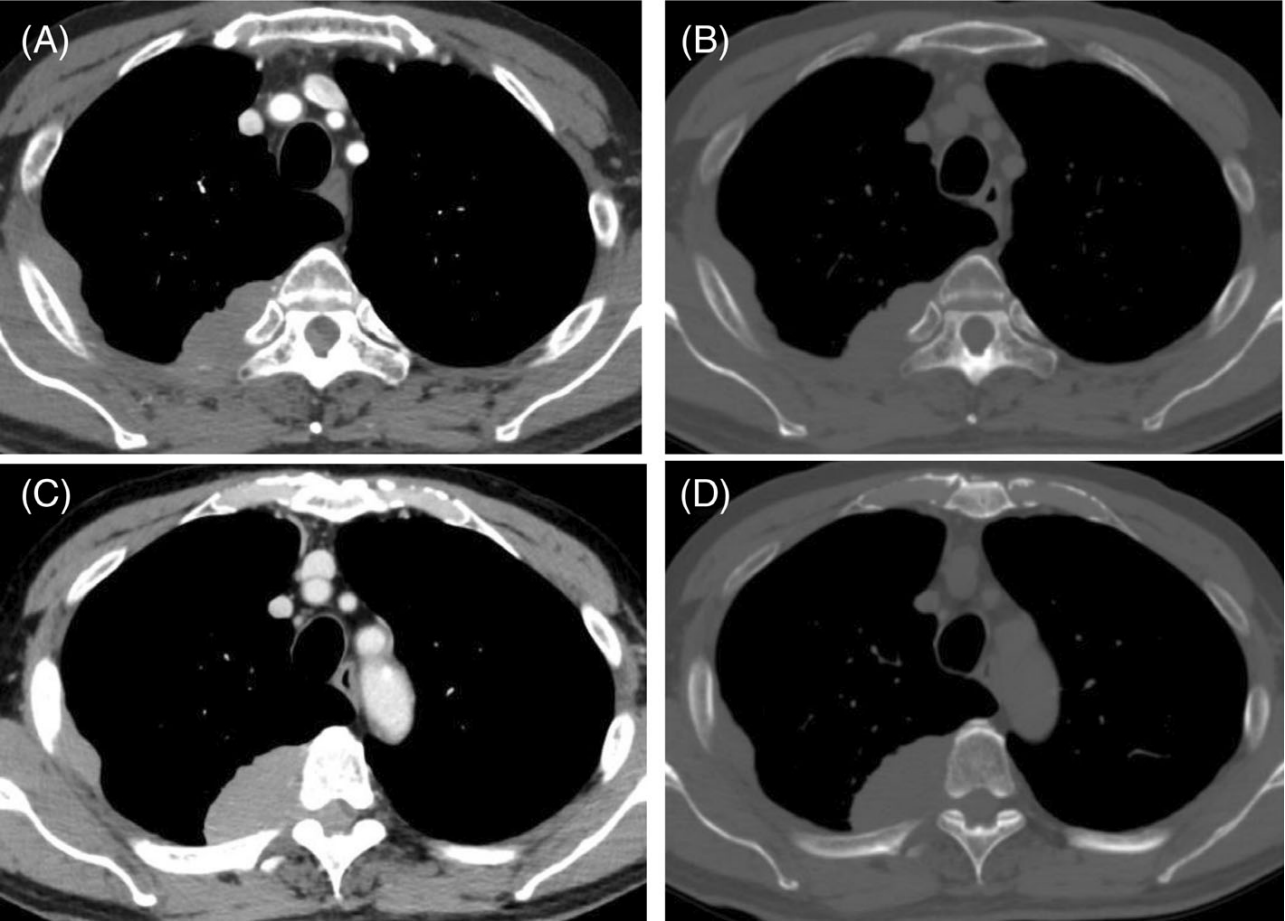
(A, B) A chest computed tomography (CT) scan showing a right chest wall mass spreading along the rib, without bone destruction, with nodular pleural thickening in the right lateral chest. The mass showing homogeneous soft tissue density, without calcification and low‐density area. (C, D) CT scan showing a mass extending from the thoracic cavity to the spinal canal at intervertebral foramen, called ‘dumbbell‐shaped lesion’.

**FIGURE 2:**
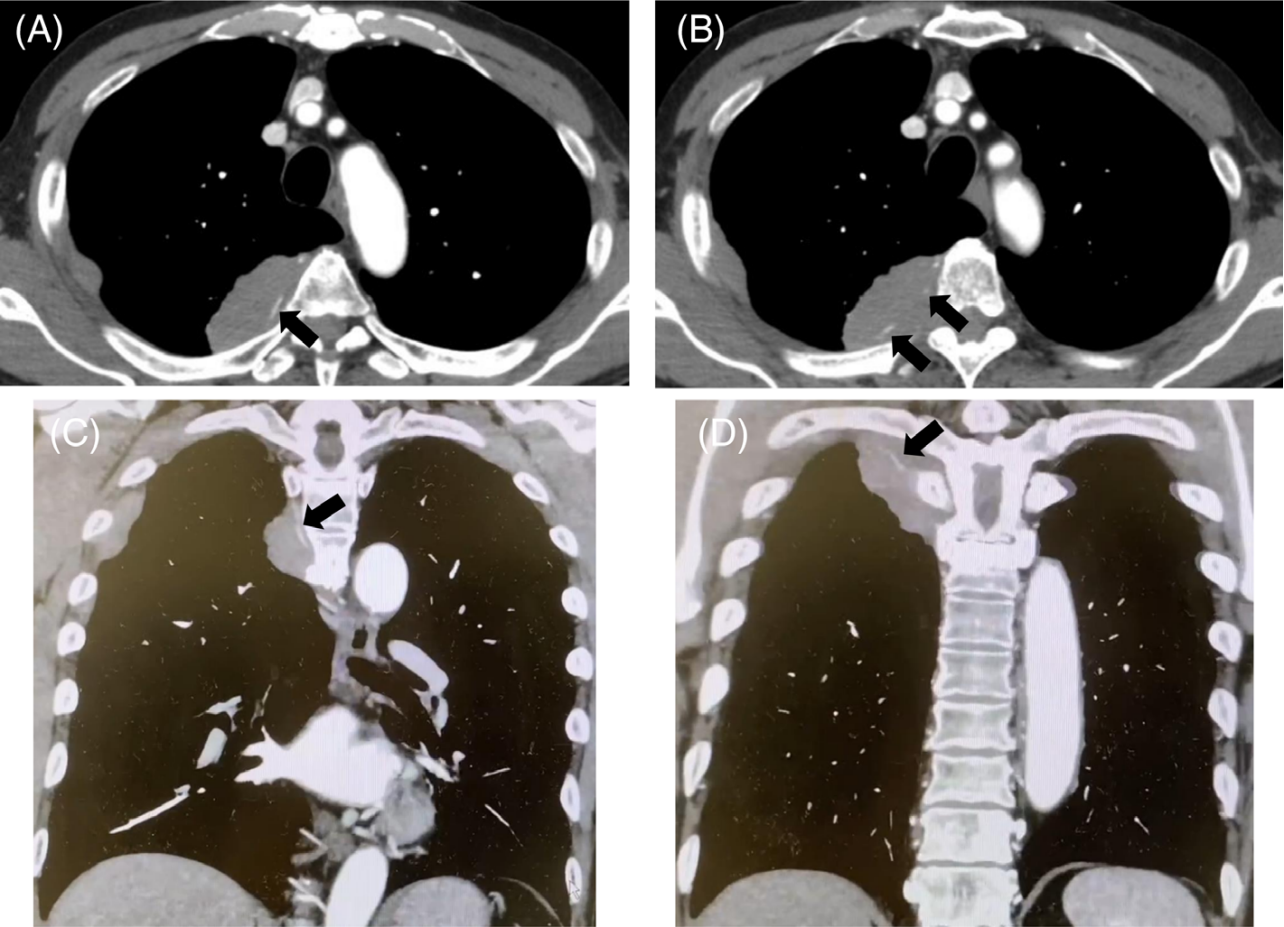
Axial (A, B) and coronal (C, D) CT images showing a right chest wall mass encasing the intercostal arteries, indicative of a positive ‘pleural sandwich sign’ (arrow).

**FIGURE 3:**
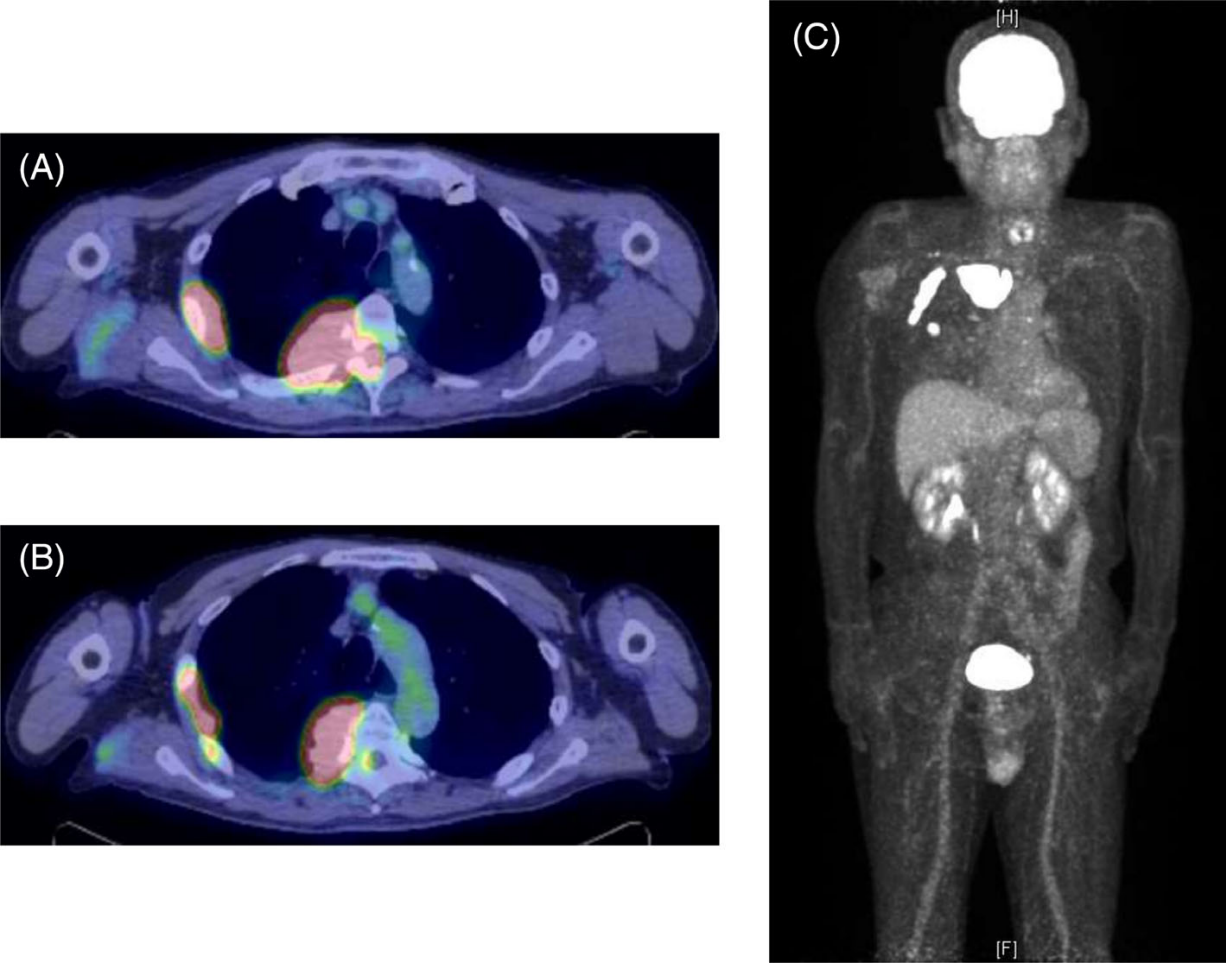
(A–C) Fluorodeoxyglucose‐positron emission tomography (FDG‐PET) scanning revealed high 18‐fluorodeoxyglucose uptake in the right chest wall without any other uptake.

A chest radiograph revealed a right tumour‐like shadow in the upper right lung field. CT revealed an irregular shaped mass in the right posterior chest wall without bone destruction. The mass was a dumbbell‐shaped lesion extending from the thoracic cavity to the spinal canal at the intervertebral foramen of the fifth thoracic vertebra (Figure 1). CT also showed that the mass enveloped the intercostal artery, demonstrating a positive ‘pleural sandwich sign’ (Figure 2). In the fluorodeoxyglucose‐positron emission tomography (FDG‐PET) scanning, the maximum standard uptake value (SUV_max_) for chest wall mass was 30.6 (Figure 3). There was no uptake except in the right lateral to posterior chest wall.

CT‐guided biopsy was performed. Based on histopathological findings, we diagnosed the tumour as a double‐expressor diffuse large B‐cell lymphoma with non‐germinal center B‐cell derivation. Since there was no evidence of other primary lesions, the patient was diagnosed to have primary chest wall lymphoma. Rituximab plus cyclophosphamide, doxorubicin, vincristine, and prednisone therapy was initiated. Tumour shrinkage was confirmed by chest radiograph, and he is continuing chemotherapy.

## DISCUSSION

Chest wall tumours mainly include mesenchymal tumours, intrathoracic epithelial malignant tumours infiltration, and metastasis of malignant tumours.^3^, ^4^, ^6^ There are limited studies on primary chest wall lymphomas in patients without prior pleural disease. Hence, clear imaging features indicative of chest wall lymphomas have not been established. However, multiple imaging findings in our patient may be suggestive of lymphoma.

Our case showed a positive ‘pleural sandwich sign’ in contrast‐enhanced CT, in which the intercostal arteries are well visualized in the conglomerated pleural and chest wall masses. This sign has been reported in three previous reports of primary chest wall lymphomas occurring in patients without preceding pleural disease.^7^, ^8^, ^9^ Kim et al. reported that this sign is not seen in non‐lymphoma pleural tumours because of their rapid invasion of intercostal vessels.^9^ This finding is likely to occur due to lymphoma infiltration to the perivascular interstitium without invading the vascular wall.^10^, ^11^

An infiltrative soft tissue mass spreading around the bone without bone destruction was also seen in our case. This feature has been reported in patients with several primary chest wall lymphoma without preceding pleural disease.^12^, ^13^ This finding is not usually seen in epithelial malignancies, because the invasion of malignant epithelial tumours is usually accompanied by bone destruction.

The appearance of a ‘dumbbell‐shaped mass’ is defined as a tumour that penetrates the intervertebral foramina and assumes an hourglass shape. Although dumbbell‐shaped tumours are a common finding in neurogenic tumours, several other mesenchymal tumours can show this finding.^14^, ^15^ Lymphomas commonly infiltrate the neurovascular bundle and extend through the subcutaneous tissues.^11^ and sometimes spread around the bone without bone destruction. Consequently, lymphomas that develop in paravertebral lesions may invade the spinal canal through the intervertebral foramen, and present with dumbbell‐shaped lesions.^16^ Therefore, this finding requires consideration of primary chest wall lymphoma as well as neurogenic tumours.

Lastly, high SUV_max_ values in chest wall tumours may help to diagnose lymphomas. Lymphomas, especially aggressive B‐cell lymphomas, often have higher SUV_max_ values, sometimes reaching 30–60.^17^, ^18^, ^19^, ^20^, ^21^ Many mesenchymal tumours of the chest wall except lymphomas have been shown to have relatively lower SUV^max^ values (10 or less).^22^, ^23^, ^24^ From these findings, higher SUV_max_ values in chest wall tumour can be helpful in differentiating lymphomas from other mesenchymal tumours.

For our case, we considered malignant lymphoma as well as neurogenic tumour, sarcoma, and solitary fibrous tumour as differential diagnoses of the chest wall tumour presenting as a dumbbell‐shaped mass. We considered benign neurogenic tumours and solitary fibrous tumours to be atypical because of their relatively low SUVmax on PET‐CT.^22^ Sarcomas were also considered atypical because most of them have been reported to be associated with bone destruction in the past.^15^ Malignant neurogenic tumours are the most difficult to differentiate from lymphomas on imaging findings because of the possibility of dumbbell‐shaped masses without bone destruction and the high SUVmax on PET‐CT in some cases.^15^, ^22^ The pleural sandwich sign has not been reported in diseases other than malignant lymphoma, and may be useful in differentiating these diseases.

In conclusion, ‘the pleural sandwich sign’, ‘infiltrative soft tissue mass spreading around the bone without bone destruction’, ‘dumbbell‐shaped mass’, and ‘high SUV_max_ values’ seen in our case are suggestive imaging findings of primary malignant lymphoma of the chest wall. This case report suggests that these findings may be helpful for diagnosing chest wall lymphomas even in patients without prior pleural disease.

## AUTHOR CONTRIBUTION

All authors contributed to the study conception. The literature search was performed and the first draft of the manuscript was written by Masanori Tanaka. The manuscript was corrected by Daichi Fujimoto. All authors commented on previous versions of the manuscript. All authors read and approved the final manuscript.

## CONFLICT OF INTEREST

None declared.

## ETHICS STATEMENT

The authors declare that appropriate written informed consent was obtained for the publication of this manuscript and accompanying images.

## Data Availability

The data that support the findings of this study are available from the corresponding author upon reasonable request.
